# Vegetation structure determines the spatial variability of soil biodiversity across biomes

**DOI:** 10.1038/s41598-020-78483-z

**Published:** 2020-12-09

**Authors:** Jorge Durán, Manuel Delgado-Baquerizo

**Affiliations:** 1 Centre for Functional Ecology, Department of Life Sciences, University of Coimbra, 3000-456 Coimbra, Portugal; 2 Departamento de Sistemas Físicos, Químicos y Naturales, Universidad Pablo de Olavide, 41013 Sevilla, Spain

**Keywords:** Biogeochemistry, Climate-change ecology, Ecosystem ecology, Microbial ecology

## Abstract

The factors controlling the spatial variability of soil biodiversity remain largely undetermined. We conducted a global field survey to evaluate how and why the within-site spatial variability of soil biodiversity (i.e. richness and community composition) changes across global biomes with contrasting soil ages, climates and vegetation types. We found that the spatial variability of bacteria, fungi, protists, and invertebrates is positively correlated across ecosystems. We also show that the spatial variability of soil biodiversity is mainly controlled by changes in vegetation structure driven by soil age and aridity. Areas with high plant cover, but low spatial heterogeneity, were associated with low levels of spatial variability in soil biodiversity. Further, our work advances the existence of significant, undescribed links between the spatial variability of soil biodiversity and key ecosystem functions. Taken together, our findings indicate that reductions in plant cover (e.g., via desertification, increases in aridity, or deforestation), are likely to increase the spatial variability of multiple soil organisms and that such changes are likely to negatively impact ecosystem functioning across global biomes.

## Introduction

The uneven distribution of soil features within a given location (hereafter spatial variability), is a ubiquitous characteristic of most terrestrial ecosystems^1,2^. A wide body of the literature reveals that the within-ecosystem spatial variability of soil properties and functions is largely controlled by the interaction of multiple biological, chemical, and physical attributes^3–5^. However, much less is known about the factors that control the spatial variability of belowground organisms. We know that soil biodiversity is an integral driver of multiple ecosystem functions^6,7^, and studies over the last decade have helped to identify the most important environmental factors controlling the diversity and community composition of soil organisms across space and time^8–11^. However, one aspect of soil biodiversity that has been neglected in these studies is its spatial variability. Thus, strikingly, very little is known about the factors controlling the within-site spatial variability of soil biodiversity across contrasting (in terms of climate and vegetation type) biomes and in soils with different soil ages.

The spatial variability of soil properties is known to play essential roles in controlling ecosystem functioning^8,9,11–13^, including plant performance and competitive ability^14^, ecosystem productivity^15^, trophic interactions^16^, and soil nutrient cycling^17,18^. Advancing our knowledge on the major patterns controlling the spatial variability of the diversity and community composition of the myriad of soil organisms including bacteria, fungi, protists and invertebrates is therefore fundamental to better understand the wide range of ecosystem processes that they control. However, we are far from understanding how the spatial variability in soil biodiversity is associated with key biotic and abiotic drivers, which limits our capacity to forecast how global environmental changes could alter not only the spatial distribution of soil organisms, but also terrestrial ecosystem functioning.

To address these knowledge gaps, we conducted soil and vegetation field surveys in six continents and 87 sites ranging in soil age from hundreds to millions of years, and encompassing a wide range of climatic conditions (tropical, temperate, continental, polar, and arid), vegetation types (grasslands, shrublands, forests, and croplands), and origins (volcanic, sedimentary, dunes, and glaciers) (Tables S1 and S2). The samples from this study were collected within 16 globally distributed soil chronosequences as described in Delgado-Baquerizo et al*.* (2019). This database has been previously used to investigate the changes in soil richness during ecosystem development, but the major ecological predictors of the spatial variability in soil biodiversity remained to be described. Thus, we used amplicon sequencing information on the diversity of bacteria, fungi, protists and invertebrates, available for five soil samples within each location, to assess the within-site spatial variability (i.e. coefficient of variation in soil organisms richness and community composition dissimilarity), and to investigate how and why (i.e. climate, soil properties and plant attributes) the spatial variability of the soil biodiversity changes in ecosystems across the planet with contrasting climate, vegetation and soil age.

## Methods

This study used the database available in Delgado-Baquerizo et al*.*^11^. In brief, soil and vegetation data were collected between 2016 and 2017 from 87 sites and 16 soil age chronosequences located in nine countries from six continents (Tables S1 and S2). A total of 435 soil samples were included in this study. These study sites cover a wide variety of vegetation (i.e. forests, shrublands, grasslands, and croplands) climate (i.e. polar, continental, temperate, tropical, and arid), soil ages (from centuries to millions of years) and soil origins (i.e. volcanic, sedimentary, dunes, and glacier). In each chronosequence stage, we surveyed a 50 m × 50 m site. In each site, plant cover was measured using the line-intercept method from data collected in three 50 m length transects spaced 25 m apart (see Maestre et al*.*^19^ for details on the standardized sampling protocol). Average of mean annual temperature and precipitation were obtained from the Worldclim database^20^.

Five composite soil samples (i.e. each composite sample was formed by five, 10 cm depth soil cores collected in the same sampling point) were randomly collected in each site. A total of 435 soil samples were included in this study. This sampling design has been successfully used in the past to estimate the spatial variability of soil attributes in global drylands^2^. The sampling was conducted during the same days within each soil chronosequence. After the collection, soils were sieved (2 mm) and separated into two portions. One portion was air-dried and used for biochemical analyses and the other one immediately frozen at − 20 °C for molecular analyses^8,21^. Soil properties were determined using standardized protocols^19^. Soil pH was measured in a 1:2.5 (dry soil mass to deionized water) suspensions with a pH meter, and soil total organic C (soil C hereafter) was determined by colorimetry after oxidation with a mixture of potassium dichromate and sulfuric acid. We selected soil pH and C because they are important environmental drivers of belowground biodiversity, change predictably during pedogenesis, and are considered good proxies of key ecosystem processes linked to soil fertility, nutrient cycling, biological productivity, and the build-up of nutrient pools^2,8,22–24^.

Soil bacteria, fungi, protists and invertebrates richness was measured via amplicon sequencing using the Illumina MiSeq platform. Ten grams of frozen soil were ground using a mortar and liquid N to homogenize soils and obtain a representative sample. Soil DNA was extracted using the Powersoil DNA Isolation Kit (MoBio Laboratories, Carlsbad, CA, USA) according to the manufacturer’s instructions. A portion of the bacterial 16S and eukaryotic 18S rRNA genes were sequenced using the 515F/806R and Euk1391f./EukBr primer sets, respectively^11,25,26^. Bioinformatic processing was performed using a combination of QIIME, USEARCH and UNOISE3. Phylotypes were identified at the 100% identity level (zero-radius OTU [zOTU] level). The zOTU abundance tables were rarefied at 501900 (bacteria via 16S rRNA gene), 2000 (fungi via 18S rRNA gene), 800 (protists via 18S rRNA gene) and 300 (invertebrates via 18S rRNA gene) sequences/sample, respectively, to ensure even sampling depth within each belowground group of organisms. The richness of soil bacteria, fungi, protists and invertebrates was determined from rarefied OTU abundance tables. Before conducting statistical modelling, we also ensured that our choice of rarefaction level, taken to maximize the number of samples in our study, was not obscuring our results. Thus, using the samples with the highest sequence/sample yield, we tested for the impact of different levels of rarefaction on belowground diversity. Importantly, we found strong, statistically significant correlations between the diversities and community compositions of soil bacteria (rarefied at 5000 vs. 18,000 sequences/sample), fungi (rarefied at 2000 vs. 10,000 sequences/sample), protists (rarefied at 800 vs. 4000 sequences/sample), and invertebrates (rarefied at 300 vs. 1800 sequences/sample), providing evidence that our choice of rarefaction level did not affect our results or conclusions. See Delgado-Baquerizo et al*.*^11^ for further details.

To estimate the within-site spatial variability of plant cover we calculated the coefficient of variation (CV) using the cover data obtained in each of three transects per site (see above). To do so for soil C, pH, and species richness of the different taxa, we calculated their CV using the five composite soil samples obtained at each site. The CV is a relative measure of heterogeneity that can accommodate variance-mean scaling, avoiding the tendency for variance to increase with the mean^27^. Therefore, it has been shown to be more useful than absolute measures of variability such as the standard deviation for comparing variability within biological properties^1,28^. We then estimated a site-level belowground spatial variability index by calculating the arithmetic mean of individual site-level CVs of the spatial variability of bacteria, fungi, protist, and invertebrate richness. To estimate the belowground community composition dissimilarity, we first calculated site-level (87 sites) Bray–Curtis dissimilarity matrices for the community composition of soil bacteria, fungi, protists and invertebrates based on the zOTU relative abundance matrices^29^. We then averaged these organism-level Bray–Curtis dissimilarity matrices at the site-level to generate a belowground community dissimilarity index.

We constructed histograms to unveil the underlaying frequency distribution of the spatial variability of belowground richness and community composition dissimilarity of soil bacteria, fungi, protists and invertebrates, as well as that of our site-level belowground richness spatial variability and community dissimilarity indices. We explored the differences among the spatial variability of the different taxa using the permutational ANOVA (PERMANOVA) approach and a posteriori permutational pairwise comparisons^30^. A maximum of 999 permutations were used to obtain pseudo-F and p-values. Then, we used Spearman Rank Correlations to explore the relationships among the spatial variability of belowground richness and community composition dissimilarity of the different taxa. Finally, to achieve a system-level understanding of the major drivers of spatial variability of belowground richness and community composition dissimilarity of soil organisms, we used structural equation modelling (SEM). In particular, we used SEM to evaluate the multiple direct and indirect effects of biotic and abiotic drivers on our indices of site-level belowground richness spatial variability and community composition dissimilarity indices. Our a priori model based on our current knowledge is available in Fig. S1. Structural equation modelling is particularly useful in large-scale correlative studies because it allows us to partition causal influences among multiple variables, and to separate the direct and indirect effects of the predictors included in the model^31^. Variables were log- or square root-transformed, when necessary, to improve linearity in the relationships. After verifying the adequate fit of our model^32^, we were free to interpret the path coefficients of the model and their associated P-values. A path coefficient is analogous to the partial correlation coefficient, and describes the strength and sign of the relationships between two variables^31^. The probability that a path coefficient differs from zero was tested using bootstrap tests^32^. The net influence that one variable had upon another was calculated by summing all direct and indirect pathways (effects) between two variables. Then we parameterized the model using our dataset and tested its overall goodness of fit. All SEM were conducted using AMOS 20 (IBM SPSS Inc., Chicago, IL, USA). Histograms, and correlation analyses were carried out with R 3.6.1 (R Core Team, Vienna, Austria). Permutational ANOVA and pairwise comparisons were carried using Primer 6 and PERMANOVA + (Prirmer-E Ltd, Plymouth, UK).

## Results

**Figure 1:**
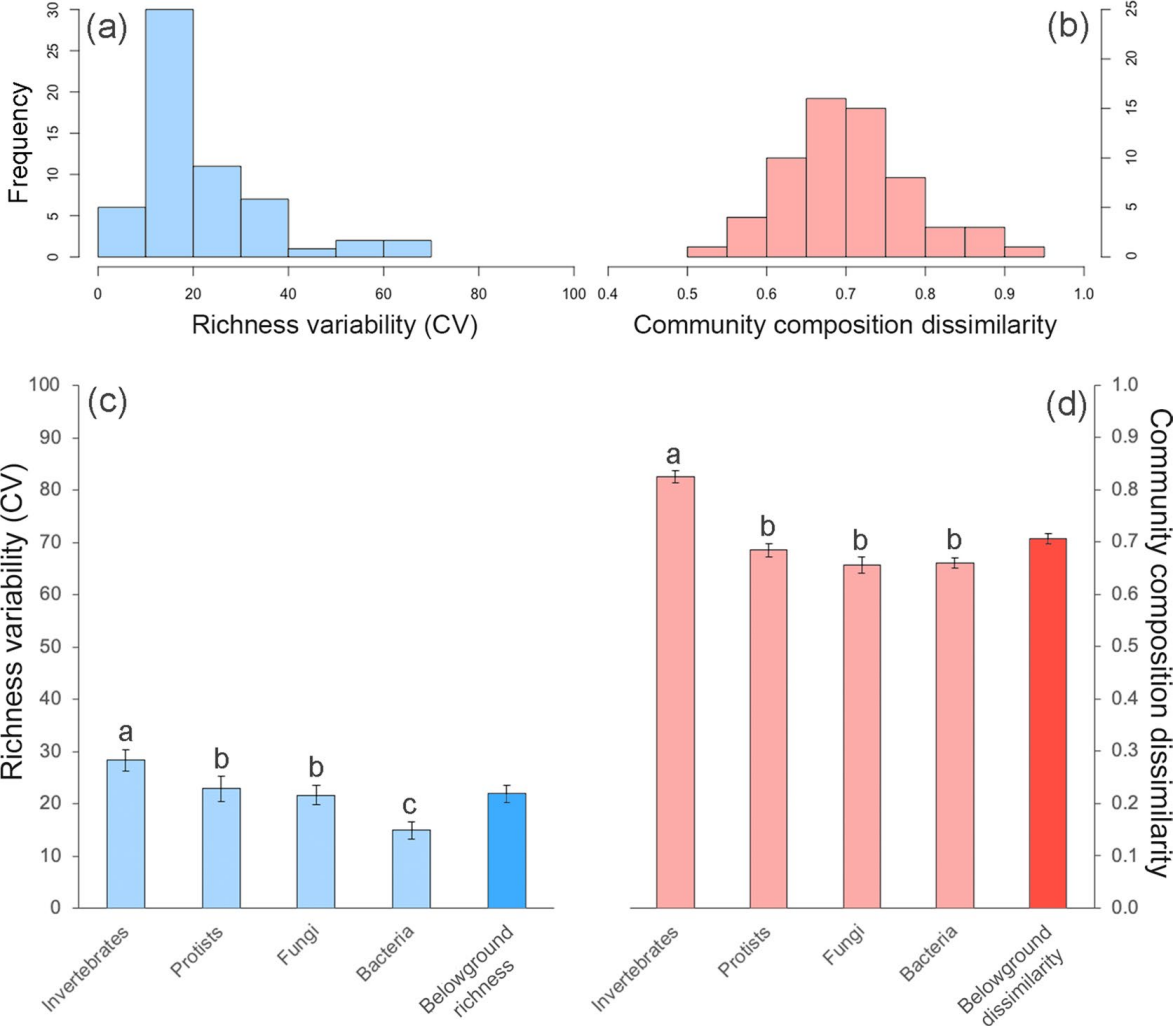
Absolute frequency histogram of the spatial variability of soil richness (coefficient of variation) (**a**) and community composition dissimilarity (**b**), as well as average values and standard errors values of the spatial variability of soil richness (**c**) and community composition dissimilarity (**d**) for the different taxa and for our indices of site-level belowground spatial variability of soil richness and community composition dissimilarity. Different letters show significant differences among taxa (permutational pairwise comparisons; P < 0.05).

**Figure 2:**
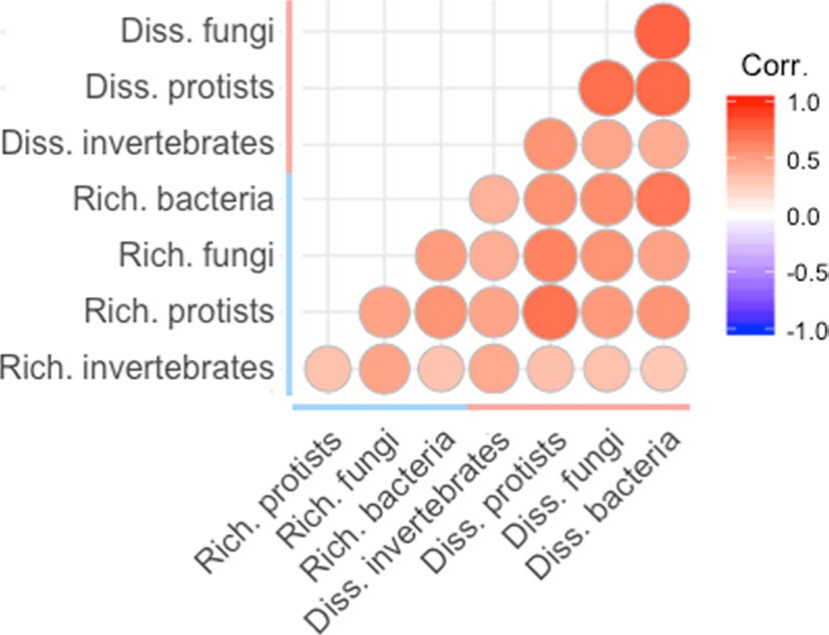
Spearman Rank significant correlations (P < 0.05) among the spatial variability of the soil richness and community composition dissimilarity of the different taxa.

We found that the within-site spatial variability of soil biodiversity is highly variable across biomes (Fig. 1). Thus, the variability of soil organism richness (via coefficient of variation) and community composition dissimilarity ranged from 1.27% to 82.12%, and from 0.44 to 0.96, respectively (Fig. 1a,b; Fig. S2). Also, whereas the spatial variability of belowground richness was relatively homogeneous across sites (except for a particularly high frequency of sites with levels of CV around 10–20%: Fig. 1a), the community composition dissimilarity followed a normal distribution (Fig. 1b; Fig. S2), suggesting that most sites have intermediate levels in the spatial variability of the community composition of soil organisms. On average, soil invertebrates showed the highest levels of within-site spatial variability (for both belowground richness and dissimilarity; Fig. 1c,d), with bacteria showing the lowest levels of variability for belowground richness (Fig. 1c), and with protists, fungi, and bacteria showing similar levels of belowground dissimilarity (Fig. 1d). Even so, the spatial variabilities of soil biodiversity for multiple soil organisms (bacteria, fungi, protists and invertebrates) were highly correlated with each other (Fig. 2) suggesting the existence of similar environmental regulators. Because of this, and for clarity, we conducted downstream analyses based on the standardized average of the spatial variability for the richness or community composition dissimilarity of all belowground taxa (i.e. our site-level belowground richness spatial variability and community dissimilarity indices; see above).

**Figure 3:**
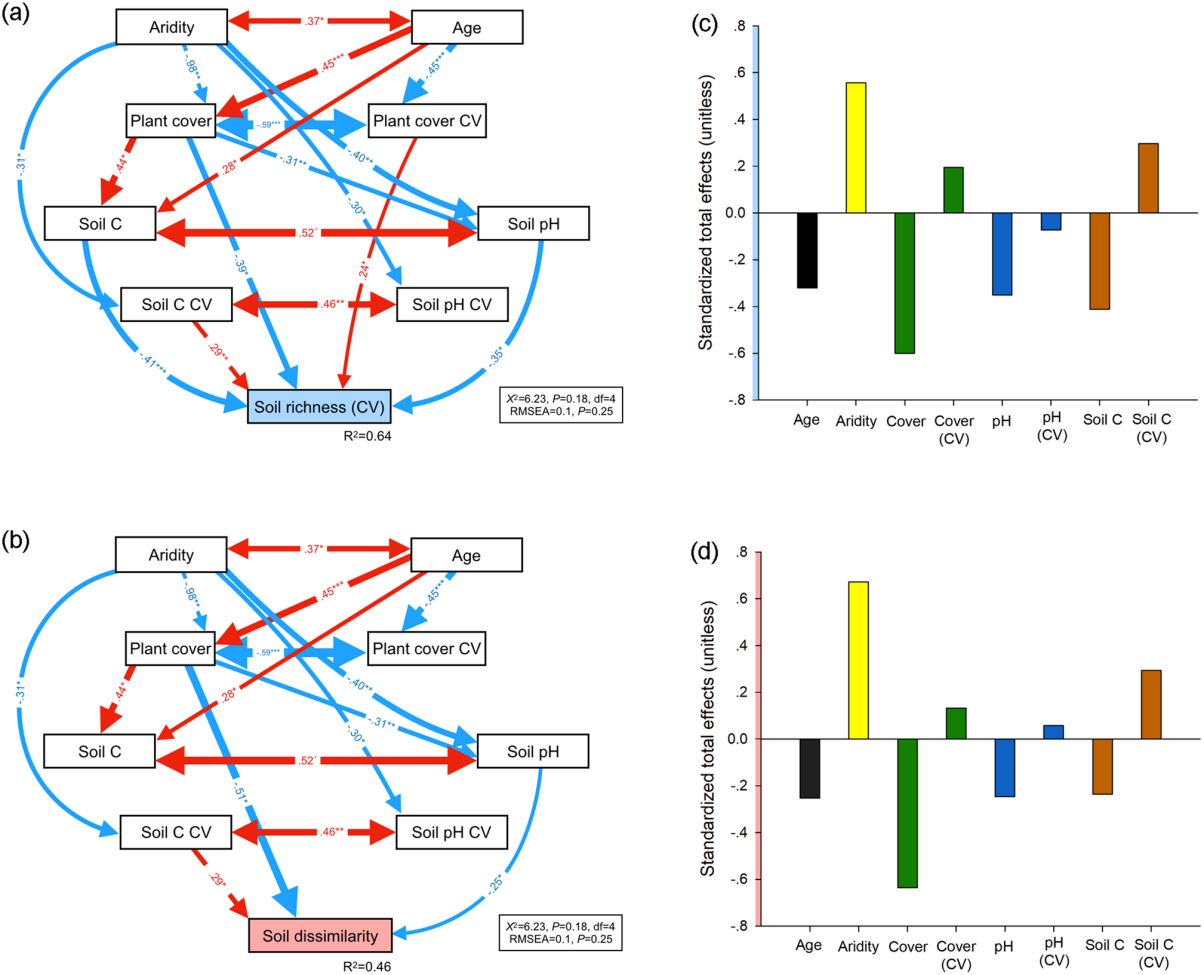
Structural equation models describing the effects of aridity, age, and plant and soil attributes on the belowground spatial variability of soil richness (**a**) and community composition dissimilarity (**b**). Numbers adjacent to arrows are standardized path coefficients, analogous to relative regression weights, and indicative of the effect size of the relationship. *P < 0.05, **P < 0.01, ***P < 0.001. Only significant relationships (P < 0.05) are shown. Red and blue arrows indicate positive and negative relationships, respectively. Arrow widths are proportional to the strength of the relationship. The proportion of variance explained (R^2^) appears alongside the response variable in the model. Goodness-of-fit statistics for each model are shown in the bottom (*df* degrees of freedom, *RMSEA* root mean squared error of approximation). Panels (**c**,**d**) show the standardized total effects (direct plus indirect effects derived from the structural equation models) of the different explanatory variables on the spatial variability of belowground richness and community composition dissimilarity, respectively.

The structural equation modelling (SEM) allowed us to identify the most important ecological predictors as well as the associations between climate, soil age, vegetation, and soil properties as drivers of the spatial variability of soil biodiversity (richness and community composition). Thus, our SEM explained 64% of the spatial variability of belowground richness (Fig. 3a) and 46% of its community composition dissimilarity (Fig. 3b). Our analyses indicate that changes in vegetation structure (i.e. increases in plant cover and decreases in its spatial variability), associated to decreases in aridity and increases in soil age, determine the spatial variability in the diversity of multiple soil organisms across global biomes (Fig. 3 and Fig. S3). Moreover, the standardized total effects (sum of direct and indirect effects from SEM) indicated that aridity (positive effect) and plant cover (negative effect) were the most important factors regulating the spatial variability of belowground richness (Fig. 3c) and community composition (Fig. 3d). Soil age, C and pH, all of them with negative total effects, were also important predictors of the belowground spatial variability, particularly for soil richness, whereas the spatial variability of soil C was an important, positive driver of the belowground richness and community composition dissimilarity.

## Discussion

This study represents the first attempt to assess how and why the within-site spatial variability of soil biodiversity (i.e. richness and community composition of multiple soil organisms) changes in terrestrial ecosystems across global biomes with contrasting climate, vegetation and soil ages. Our results provide evidence that vegetation structure, driven by changes in aridity and soil age, determines the spatial variability of soil biodiversity. It is noteworthy the unexpectedly relatively high capacity of our models to explain the variability of the spatial pattern of belowground diversity, which adds support to the relevance of the selected biotic and abiotic drivers at a global scale. Low levels of explanatory capacity is a common outcome in global surveys, in which the variability among sites is inevitably high^33^, particularly when the challenge is to characterize the variability of soil resources and belowground diversity, which can be affected simultaneously by various sources of heterogeneity that might operate at different temporal and spatial scales^28^.

Our results indicate that the spatial variability of multiple soil organisms is positively associated across biomes. In other words, we found that sites with high spatial variability for a given belowground taxa are also likely to have higher levels of spatial variability for multiple types of soil organisms including bacteria, fungi, protists and invertebrates. This result suggests that the spatial variability of different soil organisms share similar environmental drivers across contrasting biomes^11^. Also, they support the idea that soil organisms are highly related through complex networks of interactions and mutual dependencies, suggesting that disturbance-driven changes in the aboveground component of ecosystems might have cascading effects on the diversity of a broad spectrum of belowground organisms^34–36^.

Vegetation structure was identified as the main factor controlling the within-site spatial variability of multiple soil organisms. Thus, plant cover and, to a lesser extent, its spatial heterogeneity, revealed as key drivers of the spatial variability of both belowground richness and community composition. Previous studies conducted both at the local^1,37^ and global scales^2^ have shown that decreases in vegetation cover and increases in woody plant encroachment increase the spatial variability of soil resources through the development of high fertility areas under and around plant canopies (characterized by higher production of above- and below- ground litter, leachates, and exudates and lower erosion rates), and low fertility areas in the zones devoid of perennial vascular vegetation^1,18,38^. Here, we provide new insights from a cross-biome survey, that decreases in vegetation cover (and increases in its own spatial variability) are strongly and consistently linked to increases in the spatial variability of the richness and community composition of all belowground taxa analyzed. More importantly, our results show that the strong control of the vegetation cover on the spatial variability of belowground organisms can be direct, but also indirect, via changes in the levels and spatial variability of contrasting soil characteristics (e.g. pH and total C).

Our study has implications for the understanding of global change impacts on soil biodiversity and ecosystem functioning. Plant cover is known to increase in many ecosystems following ecological succession, and also to be negatively associated with aridity at the global scale^39,40^. Local and global field surveys have shown that important soil attributes associated with nutrient cycling or organic matter decomposition become more spatially heterogeneous as plant cover decreases with increasing aridity^1,2,41,42^. A previous study from the survey used here, suggested that plant cover and soil pH were the main drivers of the changes in the richness of multiple soil organisms during ecosystem development^11^. Here, we further advance that increases in aridity (as expected with climate change in coming decades) and in soil age are also important drivers of the within-site spatial variability of soil biodiversity. However, most of their effects seem to operate via changes in plant cover and structure, and concomitant changes in soil features and spatial variability. These results emphasize the need to consider not only direct, but also indirect links, when seeking to identify the major drivers of soil features and to assess how they will be influenced by environmental disturbances.

Different aspects of soil spatial variability have been shown to drive many ecosystem processes^3,4,14,15^, but predicting these effects is not trivial, particularly at a global scale. The consistent associations among climate, soil properties, plant attributes and the spatial variability of soil biodiversity suggest that some of the changes in ecosystem functioning traditionally associated with variations in climate, vegetation, or in the availability or spatial variability of soil resources may operate at least partially via changes in the spatial variability of soil biodiversity. Our results therefore highlight the need for new experiments to unveil the specific functional role of the spatial distribution of the different belowground organisms, particularly at global scales.

Taken together, our results provide evidence that the spatial variability of soil biodiversity (i.e. richness and community composition) is predictable across contrasting biomes, and that the variability of multiple soil organisms follow similar spatial patterns. We show that changes in vegetation structure, associated to soil age and aridity, determine the spatial variability of soil biodiversity. Also, our findings further advance that reductions in plant cover (e.g., via desertification, increases in aridity or deforestation) are likely to increase the spatial variability of multiple groups of soil organisms, with likely important implications for multiple soil and ecosystem functions. These findings are integral to improve our ability to fully understand and forecast the complex effects of different climate change drivers on soil biodiversity, as well as to design more effective early detection and mitigation measures.

## Supplementary Information

Supplementary Information 1.

Supplementary Information 1.

Supplementary Information 2.

Supplementary Table S1.

Supplementary Table S2.
