# Supplementary figures and images for: Vegetation structure determines the spatial variability of soil biodiversity across biomes
Source: Sci Rep. 2020 Dec 9;10:21500. doi: 10.1038/s41598-020-78483-z (PMC7725809; doi:10.1038/s41598-020-78483-z)

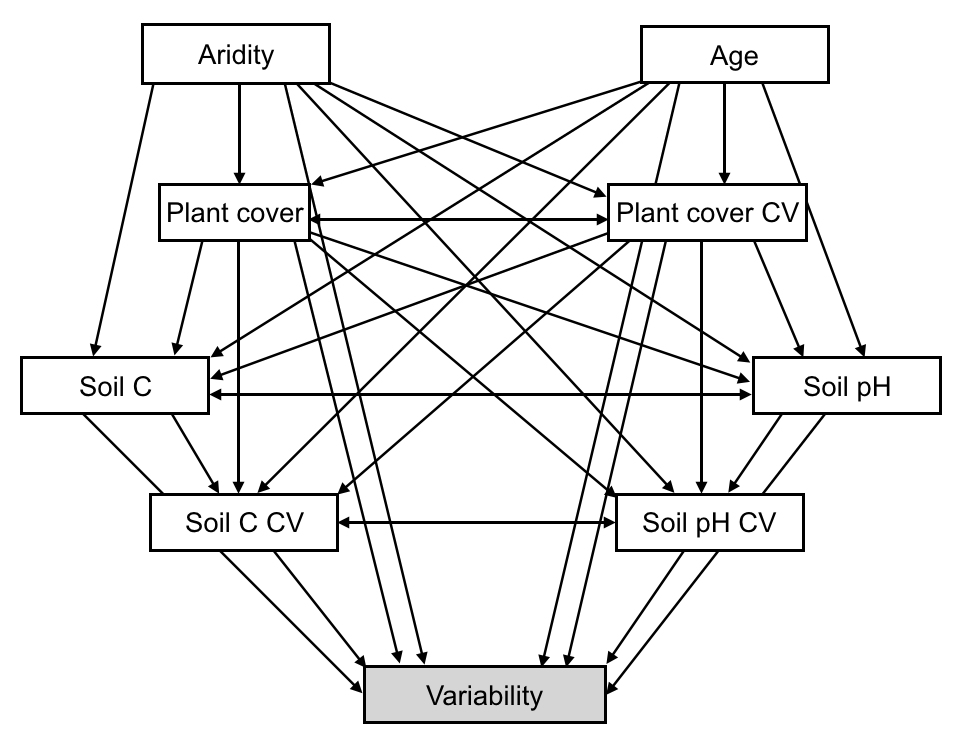

Supplement: Supplementary file 1 — Supplementary Information 1. [file 41598_2020_78483_MOESM1_ESM.png]

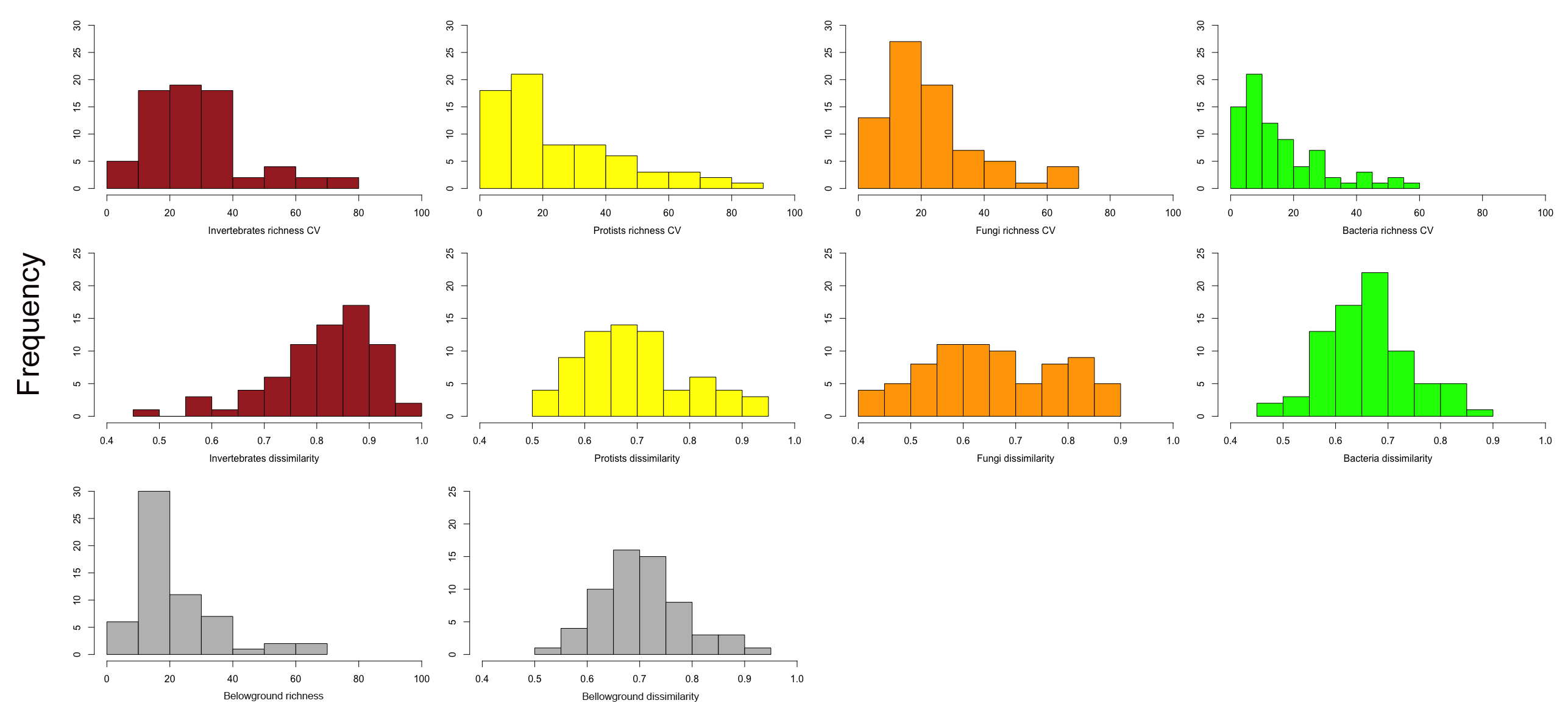

Supplement: Supplementary file 2 — Supplementary Information 1. [file 41598_2020_78483_MOESM2_ESM.tif]

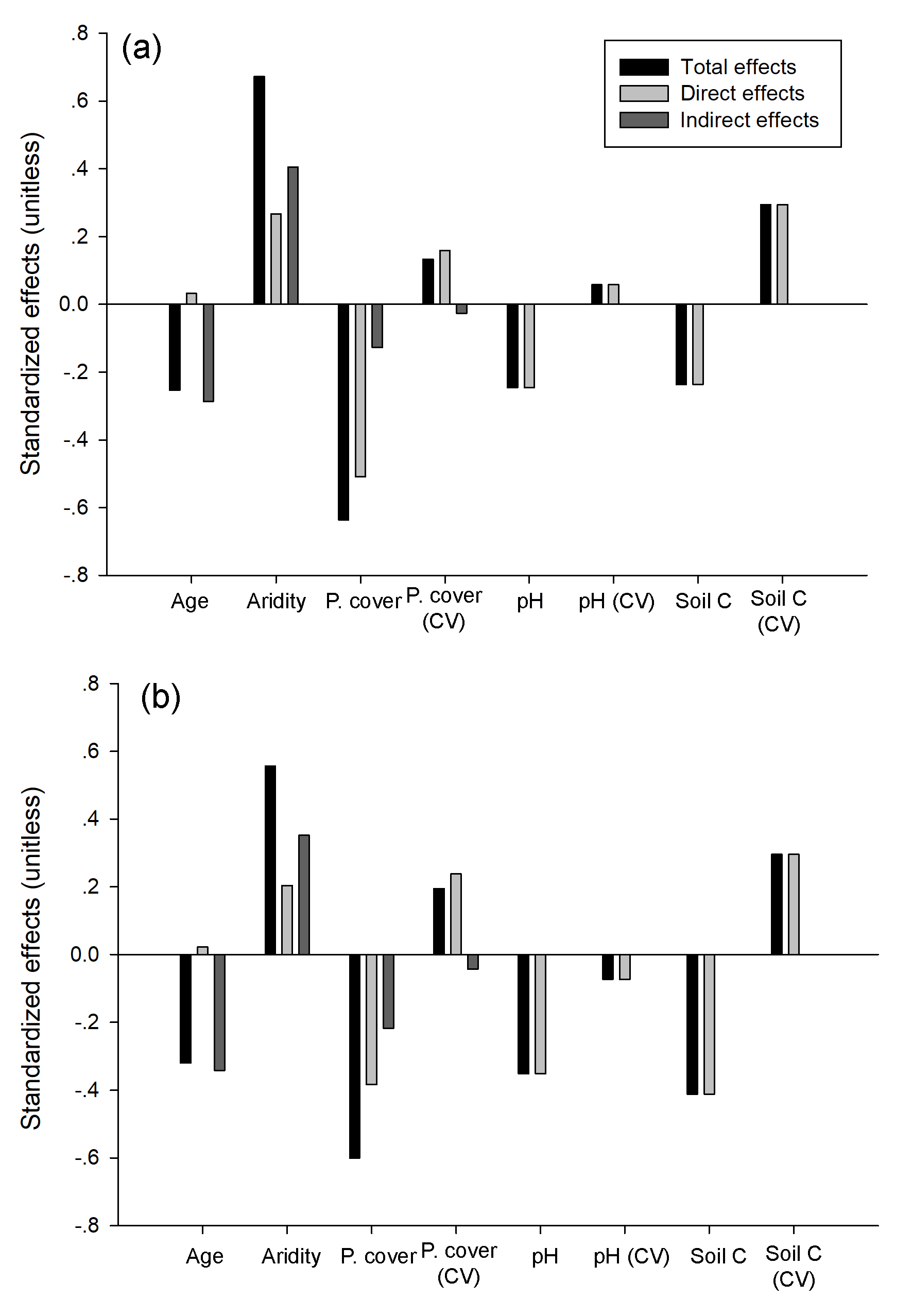

Supplement: Supplementary file 3 — Supplementary Information 2. [file 41598_2020_78483_MOESM3_ESM.tif]
