# Supplementary material for: Vegetation structure determines the spatial variability of soil biodiversity across biomes
Source: Sci Rep. 2020 Dec 9;10:21500. doi: 10.1038/s41598-020-78483-z (PMC7725809; doi:10.1038/s41598-020-78483-z)
Supplement: Supplementary file 4 — Supplementary Table S1. [file 41598_2020_78483_MOESM4_ESM.docx]

**Vegetation structure determines the spatial variability of soil biodiversity across biomes**

Jorge Durán*^1^ and Manuel Delgado-Baquerizo^2^

**Table S1.** Age, origin, aridity classification, biome type, mean annual temperature (MAT) and precipitation (MAP) for the sixteen soil chronosequences. Chronosequence origin describe the major causal agent of each chronosequence. For example, the chronosequence from ICE takes place on volcanic soils, but it is classified as a glacier chronosequence. Biome classification followed the Köppen climate classification and the major vegetation types found in our database. Aridity classification followed the Aridity Index (AI) classification: arid (0.05 > AI < 0.20), semiarid (0.20 > AI < 0.50), dry-subhumid (0.50 > AI < 0.65) and mesic (AI > 0.65).

| Label | Country | Name | Age | # of sites | Origin | Aridity classification | Biome | MAT (ºC) | MAP (mm) |
| --- | --- | --- | --- | --- | --- | --- | --- | --- | --- |
| ALPS | Austria | Alps | 0.01-120ky | 5 | Glacier | Mesic | Alpine ecosystems | 0.6 | 1182 |
| AZ | USA | SAGA | 0.9-3000ky | 4 | Volcanic | Semiarid | Arid forests | 9.68 | 427 |
| BOS | Bolivia | Cojiri | 0.025-20ky | 4 | Sedimentary | Arid | Arid shrublands | 8.4 | 141 |
| BOV | Bolivia | Chiar Kkollu | 0.025-20ky | 4 | Volcanic | Arid | Arid shrublands | 7.55 | 106 |
| CAL | USA | Merced | 0.1-3000ky | 6 | Sedimentary | Semiarid | Temperate grasslands | 16.32 | 360 |
| CH | Chile | Conguillio | 0.06-5000ky | 6 | Volcanic | Mesic | Temperate forests | 8.95 | 1917 |
| CI | Spain | La Palma | 0.5-1700ky | 7 | Volcanic | Dry-subhumid | Temperate forests | 13.27 | 507 |
| CO | USA | Coal creek | 5-2000ky | 6 | Sedimentary | Semiarid | Cold grasslands | 8.95 | 431 |
| HA | USA | Hawaii | 0.3-4100ky | 4 | Volcanic | Mesic | Tropical forests | 16.03 | 1885 |
| ICE | Iceland | Mt Hekla | 0.1-0.9ky | 5 | Glacier | Mesic | Polar moss heaths | 3.7 | 1339 |
| JOR | USA | Jornada Desert | 1.1-25ky | 4 | Sedimentary | Arid | Arid forblands | 14.65 | 265 |
| MEX | Mexico | Chichinautzin | 1-100ky | 8 | Volcanic | Mesic | Temperate forests | 11.06 | 1235 |
| MI | USA | Lake Michigan | 0.7-4ky | 10 | Sand dunes | Mesic | Cold forests | 6.1 | 774 |
| QL | Australia | Cooloola | 3.6-716ky | 6 | Sand dunes | Mesic | Temperate forests | 20.77 | 1516 |
| TA | Taiwan | Taiwan | 28-399ky | 4 | Sedimentary | Mesic | Temperate croplands | 21.33 | 2365 |
| WA | Australia | Jurien Bay | 0.1-2000ky | 6 | Sand dunes | Semiarid | Temperate shrublads | 18.97 | 557 |
