# Supplementary material for: Vegetation structure determines the spatial variability of soil biodiversity across biomes
Source: Sci Rep. 2020 Dec 9;10:21500. doi: 10.1038/s41598-020-78483-z (PMC7725809; doi:10.1038/s41598-020-78483-z)
Supplement: Supplementary file 5 — Supplementary Table S2. [file 41598_2020_78483_MOESM5_ESM.docx]

**Vegetation structure determines the spatial variability of soil biodiversity across biomes**

Jorge Durán*^1^ and Manuel Delgado-Baquerizo^2^

**Table S2.** Dominant vegetation community composition in each of the stages for the 16 soil chronosequences included in this study.

| **Name** | **Stage** | **Age (years)** | **Dominant vegetation** |
| --- | --- | --- | --- |
| ALPS | 1 | 10 | *Saxifraga azoides, Saxifraga oppositifolia, Poa alpina, Linaria alpina, Artemisia gentipi* |
|  | 2 | 45 | *Trifolium pallescens, Campanula scheuchzeri, Saxifraga oppositiolia, Saxifraga aizoides* |
|  | 3 | 125 | *Kobresia myosuroides, Agrostis alpina, Alchemilla fissa, Trifolium pratense spp., Nivale* |
|  | 4 | 10000 | *Avenula versicolor, Carex sempervirens, Festuca halleri, Anthoxanthum alpinum* |
|  | 5 | 120000 | *Fagus sylvatica, Abies alba, Acer pseudoplatanus, Picea abies, Quercus robur* |
| AZ | 1 | 900 | *Juniperus monosperma, Pinus edulis, Bouteloua gracilis* |
|  | 2 | 55000 | *Juniperus monosperma, Pinus edulis, Bouteloua gracilis* |
|  | 3 | 750000 | *Juniperus monosperma, Pinus edulis, Bouteloua gracilis* |
|  | 4 | 3000000 | *Juniperus monosperma, Pinus edulis, Bouteloua gracilis* |
| BOS | 1 | 25 | *Astragalus pusillus, Atriplex imbricata, Baccharis boliviensis, Baccharis tola, Ephedra breana, Haplopappus rigidus, Junellia seriphioides, Lycium chanar, Opuntia boliviensis* |
|  | 2 | 11400 | *Fabiana densa, Atriplex imbricata, Baccharis boliviensis, Lycium chanar, Baccharis tola, Haplopappus rigidus, Hoffmannseggia minor, Junellia seriphioides, Mutisia ledifolia, Nassella curviseta* |
|  | 3 | 14100 | *Atriplex imbricata, Baccharis boliviensis, Haplopappus rigidus, Junellia seriphioides, Lycium chanar, Mutisia ledifolia, Nassella curviseta, Trichocereus atacamensis* |
|  | 4 | 20000 | *Atriplex imbricata, Baccharis boliviensis, Cheilanthes ternifolia, Diplostephium cinereum, Ephedra breana, Fabiana densa, Lycium chanar, Mutisia ledifolia, Senecio dryophyllus, Senecio nutans, Stevia sp., Trichocereus atacamensis* |
| BOV | 1 | 25 | *Adesmia spinosa, Atriplex imbricata, Chuquiraga atacamensis. Frankenia triandra, Sisymbrium sp., Nassella curviseta* |
|  | 2 | 11400 | *Opuntia boliviensis, Acantholippia punensis, Atriplex imbricata, Chuquiraga atacamensis, Ephedra breana, Senecio dryophyllus, Sisymbrium sp.* |
|  | 3 | 14100 | *Acantholippia punensis, Adesmia spinosa, Atriplex imbricata, Chuquiraga atacamensis, Nassella curviseta* |
|  | 4 | 20000 | *Acantholippia punensis, Atriplex imbricata, Chuquiraga atacamensis, Senecio dryophyllus* |
| CAL | 1 | 100 | *Populus fremontii, Helianthus annuus, Amaranthus albus* |
|  | 2 | 3000 | *Quercus lobata, Silybum marianum, Hordeum murinum L* |
|  | 3 | 30000 | *Festuca californica* |
|  | 5 | 600000 | *Rytidosperma penicillatum* |
|  | 6 | 3000000 | *Festuca bromoides, F. myuros, Bromus hordaceous, B. diandrus* |
| CH | 1 | 60 | *Gaultheria pumila, Racomitrium lanuginosum* |
|  | 2 | 266 | *Lomatia hirsuta, Austrocedrus chilensis* |
|  | 3 | 776 | *Araucaria araucana, Nothofagus antarctica* |
|  | 4 | 3470 | *Nothofagus dombeyi, Araucaria araucana* |
|  | 5 | 60000 | *Nothofagus dombeyi, N. obliqua, N. alpina* |
|  | 6 | 5000000 | *Nothofagus dombeyi, N. alpina* |
| CI | 2 | 525 | *Pinus canariensis* |
|  | 3 | 6000 | *Pinus canariensis, Erica arborea, Pterocephalus porphyranthus* |
|  | 4 | 40000 | *Pinus canariensis, Adenocarpus viscosus, Chamaecytisus proliferus, Erica arborea* |
|  | 5 | 600000 | *Pinus canariensis, Adenocarpus viscosus* |
|  | 6 | 1100000 | *Pinus canariensis, Cistus symphytifolius* |
|  | 7 | 1700000 | *Pinus canariensis, Cistus symphytifolius* |
| CO | 1 | 5000 | *Juncus arcticus, Andropogon gerardii, Panicum virgatum* |
|  | 2 | 140000 | *Andropogon gerardii, Panicum virgatum* |
|  | 3 | 240000 | *Panicum virgatum, Poa compresa, Andropogon gerardii* |
|  | 4 | 640000 | *Chrysopsis sp, Andropogon gerardii, L. cinquefoil* |
|  | 5 | 1000000 | *Andropogon gerardii, M. Burgia, Poa compresa,* |
|  | 6 | 2000000 | *Andropogon gerardii, Poa compresa, M. Burgia* |
| HA | 1 | 300 | *Metrosideros polymorpha, Morella faya, Vaccinium calycinum, Ilex anomala, Cheirodendron trigynum, Cibotium glaucom, Hedychium gardnerianum, Isoetes sp. (grass), Coprosma sp., Myrsine lessertiana, Dicranopteris linearis, Machaerina angustifolia, Anemone hupehensis, , ,* |
|  | 2 | 20000 | *Metrosideros polymorpha, Cheirodendron trigynum, Cibotium glaucom, Cibotium menziesii, Ilex anomala, Freycinetia arborea, Astelia menziesii, Melicope clusiifolia, Vaccinium calycinum, Nephrolepis sp., Asplenium spp. (multi), Athyrium microphyllum, Ilex myrtifolia, Peperomia sp., Polypodium sp.,* |
|  | 3 | 150000 | *Metrosideros polymorpha, Cibotium glaucom, Cibotium menziesii, Hedychium gardnerianum, Vaccinium calycinum, Cheirodendron trigynum, Psidium cattleianum, Dicranopteris linearis, Asplenium sp., Melicope clusiifolia, Myrsine sandwicensis, Elaphoglossum sp, Polygonum punctatum (sic?) (water smartweed), Tibouchina herbacea, Peperomia sp., Psilotum nudum* |
|  | 4 | 4100000 | *Metrosideros polymorpha, Hedychium gardnerianum, Dicranopteris linearis, Pittosporum gayanum, Psidium cattleianum, Astelia menziesiana, Morella faya, Vaccinium meyenianum, Smilax hawaiensis, Elaphoglossum spp, Elaeocarpus bifidus, Clerodendrum sp, Alyxia oliviformis,* |
| ICE | 1 | 172 | *Racomitrium lanuginosum; Empetrum nigrum; Stereocaulon vesuvianum* |
|  | 2 | 463 | *Racomitrium lanuginosum; Empetrum nigrum; Arctostaphylos uva-ursi* |
|  | 3 | 628 | *Racomitrium lanuginosum; Betula nana; Hylocomium splendens; Empetrum nigrum; Arctostaphylos uva-ursi* |
|  | 4 | 717 | *Racomitrium lanuginosum; Salix phylicifolia; Empetrum nigrum; Hylocomium splendens* |
|  | 5 | 859 | *Racomitrium lanuginosum; Empetrum nigrum; Betula nana; Hylocomium splendens* |
| JOR | 1 | 1100-2200 | *Opuntia phaeacantha Engelm. var., Boerhavia spp., Eragrostis Lehmanniana Ness.* |
|  | 2 | 2200-7000 | *Sporobolus contractus Hitchc., Muhlenbergia Porteri Scribn., Larrea tridentata Cov., Ephedra trifurca Torr.* |
|  | 3 | 8000-15000 | *Boerhavia spp., Larrea tridentata Cov.* |
|  | 4 | 25000-75000 | *Boerhavia spp., Ephedra trifurca Torr., Erioneuron pulchellum* |
| MEX | 1 | 1000 | *Pinus montezumae, Bacharis conferta, Alnus firmifolia, Penstemon sp.* |
|  | 2 | 1835 | *Abies religiosa, Arbutus xalapensis, Pinus herrerae, Bacharis conferta, Pinus montezumae, Penstemon sp., Bacharis conferta, Buddleja sp.,* |
|  | 3 | 3800 | *Alnus firmifolia, Quercus laurina, Pinus montezumae, Pinus pseudostrobus* |
|  | 4 | 6200 | *Pinus montezumae, Alnus firmifolia* |
|  | 5 | 8000 | *Pinus montezumae, Bacharis conferta, Buddleja parviflora* |
|  | 6 | 10000 | *Pinus patula, Alnus firmifolia, Pinus montezumae, Senecio sp* |
|  | 7 | 30500 | *Pinus ayacahuite, Pinus pseudostrobus, Pinus montezumae* |
|  | 8 | 100000 | *Pinus montezumae, Abies religiosa, Quercus laurina, Penstemos sp., Bacharis conferta* |
| MI | 1 | 73 | *Amophilous breviligulata, Agropyron dasystachium, Cerisium pitheri, Arctostaphylos uva-ursi* |
|  | 2 | 113 | *Amophilous breviligulata, Agropyron dasystachium, Cerisium pitheri, Arctostaphylos uva-ursi, Schizachyrium scoparium* |
|  | 3 | 163 | *Arctostaphylos uva-ursi, Juniperus communis, Pinus strobus* |
|  | 4 | 243 | *Pteridium aquilinum, Pinus resinosa, Abies sp* |
|  | 5 | 485 | *Gaultheria procumbens, Pinus resinosa, Betula papyrifera* |
|  | 6 | 863 | *Abies balsamea, Pinus resinosa, Juniperus communis* |
|  | 7 | 1400 | *Abies balsamea, Pinus resinosa, Pinus strobus* |
|  | 8 | 2500 | *Pinus resinosa, Vaccinium myrtilloides, Gaultheria procumbens* |
|  | 9 | 3200 | *Pinus resinosa, Vaccinium myrtilloides, Gaultheria procumbens* |
|  | 10 | 4000 | *Pinus resinosa, Pinus strobus, Gaultheria procumbens* |
| QL | 1 | 3600 | *Eucalyptus tessellaris, Angophora costata, Eucalyptus intermedia, Casuarina littoralis, Melaleuca quinquenerva, Banksia integrifolia, Banksia serrata, Macrozamia spp., Acacia aulacocarpa, Acacia flavescens, Groundstorey, Cassytha paniculata, Gahnia sieberiana, Hardenbergia violacea* |
|  | 2 | 6700 | *Eucalyptus tessellaris, Angophora costata, Eucalyptus intermedia, Casuarina littoralis, Melaleuca quinquenerva, Banksia integrifolia, Banksia serrata, Macrozamia spp., Acacia aulacocarpa, Acacia flavescens, Groundstorey, Cassytha paniculata, Gahnia sieberiana, Hardenbergia violacea* |
|  | 3 | 134000 | *Eucalyptus tessellaris, Angophora costata, Eucalyptus intermedia, Casuarina littoralis, Melaleuca quinquenerva, Banksia integrifolia, Banksia serrata, Macrozamia spp., Acacia aulacocarpa, Acacia flavescens, Groundstorey, Cassytha paniculata, Gahnia sieberiana, Hardenbergia violacea* |
|  | 4 | 176000 | *Eucalyptus tessellaris, Angophora costata, Eucalyptus intermedia, Casuarina littoralis, Melaleuca quinquenerva, Banksia integrifolia, Banksia serrata, Macrozamia spp., Acacia aulacocarpa, Acacia flavescens, Groundstorey, Cassytha paniculata, Gahnia sieberiana, Hardenbergia violacea* |
|  | 5 | 324000 | *Eucalyptus tessellaris, Angophora costata, Eucalyptus intermedia, Casuarina littoralis, Melaleuca quinquenerva, Banksia integrifolia, Banksia serrata, Macrozamia spp., Acacia aulacocarpa, Acacia flavescens, Groundstorey, Cassytha paniculata, Gahnia sieberiana, Hardenbergia violacea* |
|  | 6 | 716000 | *Eucalyptus tessellaris, Angophora costata, Eucalyptus intermedia, Casuarina littoralis, Melaleuca quinquenerva, Banksia integrifolia, Banksia serrata, Macrozamia spp., Acacia aulacocarpa, Acacia flavescens, Groundstorey, Cassytha paniculata, Gahnia sieberiana, Hardenbergia violacea* |
| TA | 1 | 28000 | *Tea camellia* |
|  | 2 | 105000 | *Tea camellia* |
|  | 3 | 322000 | *Tea camellia* |
|  | 4 | 399000 | *Tea camellia* |
| WA | 1 | 100 | *Acacia cyclops, Acacia rostellifera, Scaevola crassifolia, Olearia axillaris, Spyridium globulosum* |
|  | 2 | 1000 | *Melaleuca systena, Acacia lasiocarpa, Acacia rostellifera* |
|  | 3 | 6500 | *Melaleuca systena, Acacia lasiocarpa, Acacia rostellifera* |
|  | 4 | 120000 | *Melaleuca systena, Banksia leptophylla, Calothamnus quadrifidus* |
|  | 5 | 480000 | *Banksia menziesii, Banksia attenuata, Mesomelaena pseudostygia, Hibbertia hypericoides* |
|  | 6 | 2000000 | *Banksia menziesii, Jacksonia floribunda, Banksia leptophylla* |
